# Evolutionary Hotspots of Seed Plants in Subtropical China: A Comparison With Species Diversity Hotspots of Woody Seed Plants

**DOI:** 10.3389/fgene.2018.00333

**Published:** 2018-08-20

**Authors:** Dengmei Fan, Jihong Huang, Huili Hu, Zhixia Sun, Shanmei Cheng, Yixuan Kou, Zhiyong Zhang

**Affiliations:** ^1^Laboratory of Subtropical Biodiversity, Jiangxi Agricultural University, Nanchang, China; ^2^Key Laboratory of Forest Ecology and Environment, The State Forestry and Grassland Administration, Institute of Forest Ecology, Environment and Protection, Chinese Academy of Forestry, Beijing, China; ^3^Key Laboratory of Plant Resources Conservation and Sustainable Utilization, South China Botanical Garden, Chinese Academy of Sciences, Guangzhou, China

**Keywords:** subtropical China, evolutionary hotspots, genetic landscape, species diversity, biodiversity conservation

## Abstract

Genetic diversity is a fundamental level of biodiversity. However, it is frequently neglected in conservation prioritization because intraspecific genetic diversity is difficult to measure at large scales. In this study, we synthesized population genetic or phylogeographic datasets of 33 seed plants in subtropical China into multi-species genetic landscapes. The genetic landscapes identified 18 evolutionary hotspots with high within-population genetic diversity (WGD), and among-population genetic diversity (AGD), or both. The western subtropical China is rich in AGD (possessing four major AGD hotspots), deserving a high conservation priority. We found that WGD was positively correlated with longitude, with most WGD hotspots locating in east subtropical China. The results showed that the locations of 12 of 18 evolutionary hotspots corresponded approximately to those of previously identified species diversity (SD) hotspots, however, a positive and significant correlation existed only between AGD and SD, not between WGD and SD. Therefore, spatial patterns of species richness in plants in subtropical China cannot generally be used as surrogate for their intraspecific diversity. This study identified multi-species evolutionary hotspots and correlated multi-species genetic diversity with SD across subtropical China for the first time, providing profound implications for the conservation of biodiversity in this important ecoregion.

## Introduction

To protect biodiversity within the constraints of limited conservation funding, it is essential to prioritize conservation efforts. The concept of biodiversity hotspots provides a strategy for conserving the greatest biodiversity at the least cost (Myers, 1988, 1990; Myers et al., 2000). Traditionally, this method relies mainly on species distribution patterns to determine which areas are most concentrated with species distribution, which areas have the most obvious endemism, and which areas have the most endangered species (Myers et al., 2000). Recently, phylogenetic diversity (PD) and the evolution of species have also received considerable attention as a means of identifying biodiversity hotspots due to a focus on long-term information on the evolution of different species (e.g., Forest et al., 2007; Mishler et al., 2014).

Genetic diversity within a species represents a fundamental level of biodiversity (Hughes et al., 2008) and is an approximation of the evolutionary potential of organisms (Vandergast et al., 2008). It provides the basis for phenotypic variation and adaptation, and underlies the evolutionary processes of lineage diversification and speciation that contribute to the patterns of species-, community- and ecosystem-level biodiversity evident today (Crutsinger et al., 2006; Hughes et al., 2008). However, despite increasing calls for explicit consideration of genetic diversity in conservation planning (Frankel, 1974; Smith et al., 1993; Laikre, 2010), intraspecific genetic diversity has been considered only in certain species-specific conservation programs (Mace and Purvis, 2008; Walpole et al., 2009). Until recent years, some studies proposed several approaches to map patterns of intraspecific genetic diversity across landscapes and regions with high evolutionary potential (evolutionary hotspots, i.e., regions with high within- and among- genetic diversity) (e.g., Vandergast et al., 2008; Thomassen et al., 2011; Carvalho et al., 2017). These powerful approaches provide an avenue to readily incorporate measure of evolutionary processes into GIS-based systematic prioritization and land-use planning, complementing traditional biodiversity hotspot identification that rely heavily on species richness and endemism.

China is a mega-diverse country, harboring more than 30,000 vascular plant species and about 2,340 terrestrial vertebrate species (Liu et al., 2003). Yet, China’s huge population and rapid and ongoing economic growth place biodiversity in China under serious threat (Ma et al., 2017). To promote the conservation of China’s huge biodiversity, biodiversity hotspots have been identified in terms of plant species richness (Huang et al., 2012; Li et al., 2015). However, evolutionary hotspots across multiple species, where diversification and speciation may be more likely to occur (Moritz, 2002), have never been identified across China. This situation is somewhat awkward because genetic diversity data have been accumulated rapidly and enormously during the last 20 years.

In this study, using the method of Vandergast et al. (2008), we aimed to map patterns of intraspecific genetic diversity (including within-population genetic diversity, WGD, and among-population genetic diversity, AGD, or genetic divergence) for multiple plant species to locate evolutionary hotspots throughout subtropical China except for Taiwan. Subtropical China (STC) locates in eastern mainland China between the Qinling Mts.-Huai River line (at C. 34°N) and the tropical South (≤22°N), and bordered by the Qinghai-Tibetan Plateau (ca. 98° E) in the west and the coastline in the east. It is mostly covered by subtropical evergreen broad-leaved forest (EBLF), which covers about 25% land areas of China (Zhao, 1986). This region has never been directly affected by extensive and unified ice-sheets (Shi et al., 1986), and thus served as one of the most important refuge areas for numerous Tertiary plant genera (Wu et al., 1980). The detection of conservation priorities or hotspots in this region is thus crucial for planning conservation of Chinese biodiversity. In the past two decades, the GIS-based distribution data of genetic diversity have been documented in dozens of plant species in subtropical China. This provides an unprecedented opportunity to map the distribution pattern of multiple-species genetic diversity and to identify evolutionary hotspots that may be essential for preserving the museum and cradle of flower plants (Lu et al., 2018).

In addition, as the distribution of plant species richness in China has been reported in previous studies (e.g., Huang et al., 2012), it is feasible to empirically test the hypothesis of species-genetic diversity correlations (SGDCs) across subtropical China. A positive correlation between species diversity (SD) and multiple-species genetic diversity may provide potential for simultaneous conservation of both SD and genetic diversity, and this also means that species richness can be taken as a surrogate of genetic diversity in conservation planning (Kahilainen et al., 2014).

## Materials and Methods

### Collecting Genetic Data

The genetic datasets for 33 different plant species distributing in subtropical region of mainland China were gathered from published literature or dissertations (permitted by the authors) from 2006 to 2016 (**Table 1**), while limiting inclusion to studies that sampled at least 10 populations in STC. For the included studies, populations less than three individuals were excluded from further analyses to reduce parameter bias due to small population size. The resulting datasets included 15 deciduous broadleaved trees, eight evergreen broadleaved trees, five perennial herbs, two conifers, two perennial deciduous vines and one annual herb. These species vary in habitat preference and ecology, including not only rare and endangered species but also dominant trees in EBLF of subtropical China.

**Table 1:** List of studies used in the genetic landscape analysis.

Life form/Taxon	Reference	Markers	Number of populations
**Deciduous broadleaved tree**			
*Cercidiphyllum japonicum*	Qi et al., 2012	cpDNA	26
*Cyclocarya paliurus*	Kou et al., 2016	cpDNA	53
*Davidia involucrata*	Chen et al., 2015; Ma et al., 2015	cpDNA, nSSR	32 (22)
*Dipteronia sinensis*	Yang et al., 2008	cpSSR	21
*Emmenopterys henryi*	Zhang et al., 2016	cpDNA, ITS	37(36)
*Euptelea pleiosperma*	Cao et al., 2016	cpDNA, ITS	25(24)
*Eurycorymbus cavaleriei*	Wang et al., 2009	cpDNA, nSSR	17(16)
*Fagus engleriana*	Lei et al., 2012	cpDNA	25
*Fagus longipetiolata*	Zhang et al., 2013	cpDNA	28
*Fagus lucida*	Zhang et al., 2013	cpDNA	21
*Ginkgo biloba*	Gong et al., 2008	cpDNA, nSSR	21 (23)
*Liriodendron chinensis*	Li, 2013; Li et al., 2014	cpDNA, nSSR	22 (12)
*Pteroceltis tatarinowii*	Li et al., 2012	cpDNA	23
*Tetracentron sinense*	Sun et al., 2014b	cpDNA	27
*Quercus variabilis*	Chen et al., 2012	cpDNA	24
**Evergreen broadleaved tree**			
*Castanopsis eyrei*	Shi et al., 2014	cpDNA, nSSR	29 (26)
*Castanopsis fargesii*	Sun et al., 2014a	cpSSR	27
*Castanopsis hystrix*	Li et al., 2007	cpSSR	16
*Castanopsis tibetana*	Fan et al., 2016	cpDNA	43
*Loropetalum chinense*	Gong et al., 2016	cpDNA	48
*Machilus thunbergii*	Fan et al., 2016	cpDNA	46
*Quercus glauca*	Xu et al., 2014	cpDNA	39
*Schima superba*	Fan et al., 2016	cpDNA	52
**Perennial herb**			
*Dysosma versipellis*	Qiu et al., 2009a	cpDNA	10
*Eomecon chionantha*	Tian, 2016	cpDNA nSSR, ITS	43 (33, 28)
*Ligularia hodgsonii*	Wang et al., 2013	cpDNA	23
*Miscanthus sinensis*	Zhao et al., 2013	nSSR	16
*Saruma henryi*	Zhou et al., 2010	cpDNA, cpSSR	16 (16)
**Conifer**			
*Cathaya argyrophylla*	Wang and Ge, 2006	nDNA	12
*Taxus wallichiana*	Gao et al., 2007; Zhang and Zhou, 2013	cpDNA, nSSR	38(13)
**Perennial deciduous vine**			
*Sargentodoxa cuneata*	Tian et al., 2015	cpDNA	81
*Tetrastigma hemsleyanum*	Wang et al., 2015	cpDNA, nSSR	19 (19)
**annual herb**			
*Glycine soja*	He, 2013	nSSR	16

*Molecular methods and number of investigated populations used in this study are indicated*.

Genetic data consisted mainly of chloroplast data, including sequence data from 27 species and chloroplast microsatellite data from four species. In addition, nuclear sequence data or nuclear microsatellite data were available for 12 species (**Table 1**). The other genetic markers, e.g., AFLP, RAPD and ISSR, have been applied much less frequently in previous phylogeographical studies of STC plants (e.g., *Dysosma versipellis*, Guan et al., 2010; *Kirengeshoma*, Qiu et al., 2009b), and thus these types of datasets were not included in this study. For each sequence dataset, we used the program ARLEQUIN 3.0 (Excoffier et al., 2005) to calculate nucleotide diversity (*π*) and haplotype diversity (*H_d_*), as well as pairwise population differentiation (*F_ST_*). For microsatellite data, expected heterozygosity (*H_E_*) were estimated in GENALEX 6 (Peakall and Smouse, 2006) and pairwise population differentiation (*F_ST_*) were calculated in ARLEQUIN 3.0 (Excoffier et al., 2005). If these statistics were available in the published articles or could be obtained from authors, they were directly used for subsequent analyses. To make within-population genetic diversity and genetic divergence measurements comparable among species and multiple loci, we normalized the estimates within species by dividing each raw estimate by the maximum value taken by the estimate in the study used.

### Identification of Genetic Diversity Hotspots

The statistics *F_ST_* were used to estimate genetic divergence among populations (i.e., among-population genetic diversity) and create divergence landscape. The estimates were firstly used to map a single species genetic divergence landscape for each species using ‘Genetic Landscapes Toolbox’ in ArcGIS 10.0 as follows. Pairwise genetic divergence values were mapped to the midpoints between collection locations. A surface was interpolated from the midpoints using Inverse Distance Weighted (IDW) interpolation (power = 2, variable search radius with 12 points, grid cell size 1 km^2^). Then the genetic landscapes for all taxa were averaged into a multiple species genetic landscape to highlight areas of congruence. The final map was divided into classes by the classification method of standard deviation, which had a middle class centered on the mean with a range of 1 standard deviation (0.5 standard deviation to either side of the mean) (Brewer and Pickle, 2002). Regions with average genetic divergence values greater than 1.5 standard deviations from the mean were defined as the divergence ‘hotspots’ (Vandergast et al., 2008).

Within-population genetic diversity was calculated in two ways. *H_d_* for sequence data and *H_E_* for microsatellite data are equivalent for both estimate the probability that two randomly chosen alleles or genotypes are different for diploid organisms (Vellend and Geber, 2005). These two statistics were here referred to as “gene diversity”. *π* is the average sequence divergence among individuals and was calculated as “sequence diversity” for sequence data. Then the value of gene diversity (*H_d_*/*H_E_*) and sequence diversity (*π*) within a population was used to create diversity landscapes, respectively. Using IDW interpolation as described above, we firstly created gene diversity or sequence diversity landscapes for each species. Diversity values (*H_d_*/*H_E_* or *π*) were mapped to the actual collection locations, rather than to the midpoints between locations as done in divergence mapping. Finally, we calculated the average diversity multi-species genetic landscape for the two datasets, respectively. Based on the range of values present, we considered the areas with gene diversity or sequence diversity values greater than 1.5 standard deviations from the mean as the diversity hotspots.

To facilitate comparison between GD hotspots and SD hotspots, each of the above three genetic landscapes was clipped to the spatial extent of subtropical evergreen broadleaved forest in mainland China (Zhang et al., 2007). Finally, we unified the three hotspot layers in an explicit landscape. Generally, separate hotspot patches were numbered as different ones. Sometimes patches with close geographical proximity were combined into one hotspot for narrative convenience.

### Species Diversity and Genetic Diversity Correlation Analyses

Species diversity data from Huang et al. (2012) include five indices, i.e., endemic richness (ER), weighted endemism (WE), PD, phylogenetic endemism (PE) and biogeographically weighted evolutionary distinctiveness (BED). As these measurements were available in the unit of county, we average the normalized indices of among-population genetic diversity (AGD, i.e., genetic divergence) and within-population genetic diversity (WGD, i.e., gene diversity and sequence diversity) in each county. Then, we used Pearson’s correlation test to compare AGD and WGD with SD across counties. We also used a linear regression analysis to correlate AGD, WGD and SD with latitude and longitude, respectively. All statistical analyses were performed using SPSS version 14.0 (SPSS Incorporation, 2005).

## Results

### GD Hotspots Based on Multiple-Species Genetic Landscapes

**FIGURE 1:**
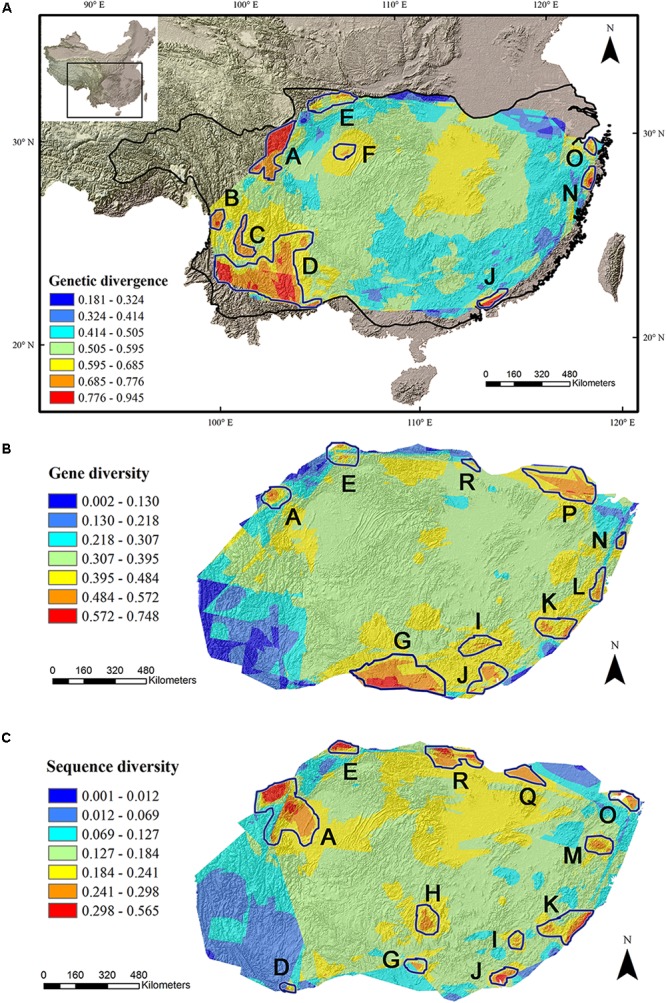
Multi-species genetic landscape for genetic divergence **(A)**, gene diversity **(B)** and sequence diversity **(C)** across subtropical China. The three genetic diversity indices range from its highest values in red to its lowest values in blue. By comparing the hotspots among the three landscapes, those hotspots belonging to the same geographic area were numbered identically. The black line in **(A)** shows the boundary of subtropical evergreen broadleaved forest in mainland China (adapted from Zhang et al., 2007).

The average divergence landscape covered 2,115,705 km^2^ after clipping to the eco-region boundary. The mean normalized divergence value was 0.549 (*SD* = 0.091) and ranged from 0.181 to 0.945 (**Figure 1A**). The areas with highest divergence (categorized as greater than 1.5 standard deviations above the mean) encompassed 135,141 km^2^ or 6.4% of the analyzed area. Nine regions were identified as AGD hotspots with highest levels of genetic divergence (**Table 2**, labeled regions A-F, J, N, and O in **Figure 1A**).

**Table 2:** Evolutionary hotspots found in the present study and location descriptions, and their concordance from species diversity hotspots of woody seed plants (Huang et al., 2012) identified in subtropical China.

Hotspot code	Location	Description	Hotspot type	Species diversity hotspots
A	North Hengduan Mountains	Min Mountains		
	Central Hengduan Mountains	Daxue Mountains, Hills of west Sichuan Basin	¶†‡	1
B	West Hengduan Mountains	Gaoligong Mountains	¶	1
C	South Hengduan Mountains	Yunling Mountains	¶	1
D	Yunnan Plateau	Wuliang Mountains, Ailao Mountains, Wumeng Mountains	¶‡	3
E	Qinling Mountain	Qinling Mountains	¶†‡	10
F	North-east Sichuan	Daba Mountains	¶	
G	Central Guangxi	Guangxi basin	†‡	
H	North Guangxi and south-west Hunan	Xuefeng Mountains, Mao’er Mountains	‡	4
I	North Guangdong and South Jiangxi	Nanling Mountains	†‡	5
J	Central Guangdong	Luofu Mountains, Jiulian Mountains	¶†‡	15
K	South Fujian	Hills of south Fujian	†‡	17
L	Northeast Fujian	Hills of northeast Fujian	†	
M	Southwest Zhejiang	Xianxialing Mountains	‡	9
N	East Zhejiang	Yandang Mountains	¶†	
O	Northeast Zhejiang	Siming Mountains	¶‡	
P	Southeast Anhui and northwest Zhejiang	Tianmu Mountains, Huang Mountains	†	8
Q	West Anhui and east Henan	Tongbai Mountains, Dahong Mountains	‡	
R	West Henan	Funiu Mountains	†‡	11

**FIGURE 2:**
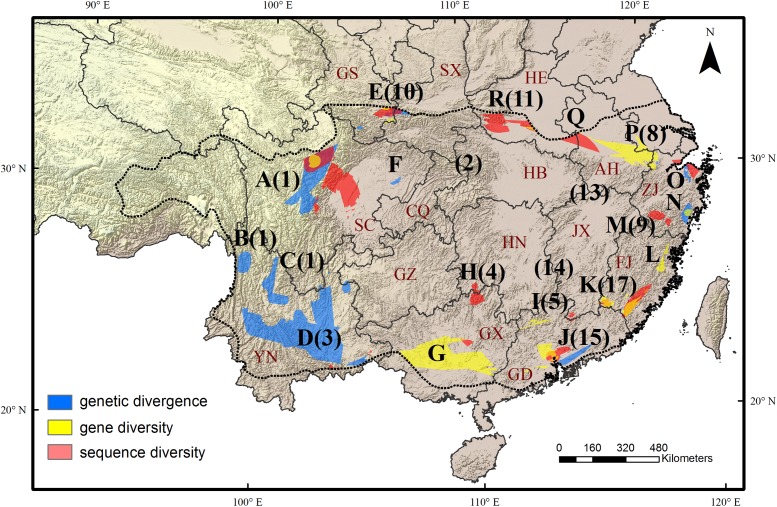
Eighteen evolutionary hotspots of genetic divergence, gene diversity and sequence diversity in subtropical China were represented by uppercase letters. The black numbers in brackets show the species diversity hotspots identified by Huang et al. (2012). The dotted line shows the boundary of subtropical evergreen broadleaved forest in mainland China (adapted from Zhang et al., 2007). The black lines show the province boundary: AH, Anhui; CQ, Chongqing; FJ, Fujian; GD, Guangdong; GS, Gansu; GX, Guangxi; GZ, Guizhou; HB, Hubei; HE, Henan; HN, Hunan; SC, Sichuan; SX, Shaanxi; ZJ, Zhejiang.

*Codes follow **Figure 2**. ^¶^, genetic divergence hotspots; ^†^, gene diversity hotspots; ^‡^, sequence diversity hotspots*.

Gene diversity (*H_d_*/*H_E_*) was averaged across all 33 species. The scaled gene diversity multi-species landscape encompassed 2,146,124 km^2^ after clipping. The mean normalized gene diversity was 0.347 (*SD* = 0.086) and ranged from 0.002 to 0.748 (**Figure 1B**). The areas with highest gene diversity covered 80,767 km^2^ or 3.8% of the study area. We identified 10 locations as WGD hotspots with highest levels of gene diversity (**Table 2**, labeled regions A, E, G, I-L, N, P, and R in **Figure 1B**).

Sequence diversity (*π*) was averaged across 28 species. The scaled sequence diversity multi-species landscape covered 2,120,862 km^2^ after clipping. The mean normalized sequence diversity value was 0.150 (*SD* = 0.055) and ranged from 0.001 to 0.565 (**Figure 1C**). The areas with highest sequence diversity encompassed 817,63 km^2^ or 3.9% of the analyzed area. We identified 12 locations as WGD hotspots with highest levels of sequence diversity (**Table 2**, labeled regions A, D, E, G-K, M, O, Q and R in **Figure 1C**). Seven of these sequence diversity hotspots overlapped with regions of high gene diversity (**Table 2** and **Figure 2**).

### Distributional Pattern of Genetic Diversity and Species Diversity

There was a general and significant west-east trend of decreasing SD in STC (**Table 3**) for the five indices used in Huang et al. (2012) (*r* = -0.280, -0.215, -0.270, -0.213, and -0.214, respectively, *p* < 0.001, **Table 3**). The spatial pattern of among-population diversity (AGD) was similar. We observed a significant west-east trend of decreasing AGD in STC region (*r* = -0.438, *p* < 0.001, **Table 3**) for normalized average genetic divergence. On the contrary, there was a significant west-east trend of increasing within-population diversity (WGD) for normalized gene diversity (*r* = 0.346, *p* < 0.001, **Table 3**) and normalized sequence diversity (*r* = 0.113, *p* < 0.001, **Table 3**).

**Table 3:** Pearson’s correlation coefficient between longitude, latitude and three GD indices (genetic divergence, gene diversity and sequence diversity) and five SD indices measured by Huang et al. (2012), i.e., endemic richness (ER), weighted endemism (WE), phylogenetic diversity (PD), phylogenetic endemism (PE), and biogeographically weighted evolutionary distinctiveness (BED).

Diversity indices	Longitude		Latitude	
	*r*	*P*	*r*	*p*
SD				
ER	-0.280^∗∗^	0.000	-0.013	0.690
WE	-0.215^∗∗^	0.000	-0.182^∗∗^	0.000
PD	-0.270^∗∗^	0.000	-0.032	0.305
PE	-0.213^∗∗^	0.000	-0.071^∗^	0.024
BED	-0.214^∗∗^	0.000	-0.212^∗∗^	0.000
GD				
Genetic divergence	-0.438^∗∗^	0.000	-0.080^∗^	0.011
Gene diversity	0.346^∗∗^	0.000	-0.017	0.591
Sequence diversity	0.113^∗∗^	0.000	0.285^∗∗^	0.000

*^∗∗^ or ^∗^ show correlation is significant at 0.01 or 0.05 level*.

There also was a significant south-north trend of decrease of AGD (*r* = -0.08, *p* < 0.05, **Table 3**). However, the latitudinal trend of WGD was positive and highly significant for sequence diversity (*r* = 0.285, *p* < 0.001, **Table 3**) but negative and not significant for gene diversity (*r* = -0.017, *p* > 0.05, **Table 3**).

### Relationship Between Genetic Diversity and Species Diversity

We found a strong biogeographical congruence between the areas with highest genetic diversity and the major biodiversity areas of the STC region. Geographically, 12 of 18 hotspots correspond approximately to the previously identified SD hotspots (**Table 2** and **Figure 2**).

**FIGURE 3:**
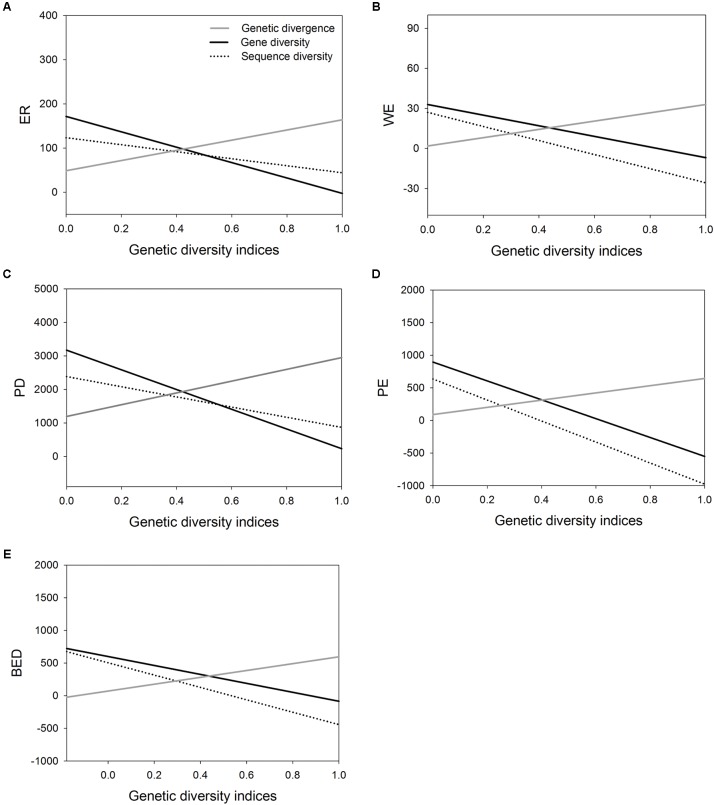
The correlations of three genetic diversity indices (genetic divergence, gene diversity and sequence diversity) with five species diversity indices measured by Huang et al. (2012). **(A)**, endemic richness (ER); **(B)**, weighted endemism (WE); **(C)**, phylogenetic diversity (PD); **(D)**, phylogenetic endemism (PE); **(E)**, biogeographically weighted evolutionary distinctiveness (BED).

Correlation analysis showed the value of AGD increases significantly with increasing value of five indices of SD, respectively (**Table 4** and **Figures 3A–E**). However, the correlation between WGD and SD was significantly negative because the value of regional average gene diversity decreased significantly with five SD indices, respectively (**Table 4** and **Figures 3A–E**), and the relationship between regional average sequence diversity and three SD indices, i.e., WE, PE and BED, was also significantly negative (**Table 4** and **Figures 3B,D,E**).

**Table 4:** The coefficient and significance values of correlation between three genetic diversity indices (genetic divergence, gene diversity and sequence diversity) and five species diversity indices measured by Huang et al. (2012), i.e., ER, WE, PD, PE, and BED.

Species diversity indices	Genetic divergence		Gene diversity		Sequence diversity	
	*r*	*p*	*r*	*p*	*r*	*p*
ER	0.77^∗^	0.014	-0.111^∗∗^	0.000	-0.32	0.308
WE	0.087^∗∗^	0.005	-0.103^∗∗^	0.001	-0.086^∗∗^	0.006
PD	0.067^∗^	0.032	-0.107^∗∗^	0.001	-0.035	0.264
PE	0.068^∗^	0.031	-0.168^∗∗^	0.000	-0.118^∗∗^	0.000
BED	0.083^∗∗^	0.008	-0.100^∗∗^	0.001	-0.087^∗∗^	0.005

*^∗∗^ or ^∗^ show correlation is significant at 0.01 or 0.05 level*.

## Discussion

### Patterns of Among-Population and Within-Population Genetic Diversity

Our multi-species genetic landscapes revealed 9 hotspots where among-population genetic diversity (or genetic divergence) is consistently high, 10 gene diversity hotspots and 12 sequence diversity hotspots where within-population genetic diversity is high. These hotspots were grouped into 18 geographic areas and most distributed at the periphery of subtropical China (**Table 2** and **Figure 2**). Three hotspots have consistently high values of genetic divergence, gene and sequence diversity (A-North and central Hengduan; E-Qinling; J-Central Guangdong). These three mountainous regions are the important famous geographic division in China’s topography and phytogeography (Li and Li, 1997; Zhu, 2013; Zhao et al., 2014). In addition, other evolutionary hotspots mostly located in the transitional zones between different vegetation and climate. For instance, the mountainous areas of north STC (hotspots E, O-R) and south STC (G-J) lie in the buffer zones between subtropical climate and warm temperate climate, tropical climate, respectively. These areas were buffered against climate extreme and have been less affected by past environmental changes than the other areas, which are prone to have high contemporary diversity and endemism (Fjeldså and Lovett, 1997). Similar patterns of periphery evolutionary hotspots have also been found in other regions of the world, such as the southern California (Vandergast et al., 2008), and the Mojave Desert (Vandergast et al., 2013).

The same as SD of Huang et al. (2012), we found that AGD decreases significantly from west to east. Most notably, four large AGD hotspots lie in the areas along Hengduan Mountains (A-C) and Yunnan Plateau (D) (**Figure 1A**), both of them feature a high level of orographic heterogeneity and has several physical barriers that restrict east-west plant migration and genetic interchange, thereby facilitating genetic differentiation among populations. For example, three of the four hotspots (A, C, and D) are transected by the ‘Tanaka-Kaiyong Line’ (TKL), a major phytogeographic boundary in southwest China separating Sino-Himalayan and Sino-Japanese Floras of East Asia (Li and Li, 1997). Several phylogeographic studies demonstrated that the TKL impeded genetic exchange between populations of the two sides (Fan et al., 2013; Zhao and Gong, 2015; Zhao et al., 2018), which would have contributed to high genetic divergence in these regions.

In contrast, east STC harbors much lower levels of AGD (**Figure 1A**), which could be interpreted by fewer physical barriers to gene flow. China is well-known with its three-step terrain from the west to the east. Although there are numerous mountains or hills in east China, they seldom exceed 2000 m above sea level and thus rarely function as barriers (but see Sun et al., 2014a). On the contrary, many mountain ranges (such as the Nanling Mountains, the Wuyi Mountains and the Luoxiao Mountains) in east STC played a role as dispersal corridors during the Quaternary climate oscillations, facilitating gene exchange among isolated populations (Fan et al., 2017; Tian et al., 2018). In addition, when those dispersal corridors facilitated inter-/postglacial range expansions, a south-north decreasing trend of AGD might be observed because the lower genetic divergence at higher latitudes may result from a genetic drift or bottleneck effect during recolonization from refugia.

Both WGD parameters, gene diversity and sequence diversity, are correlated with longitude positively and significantly and most WGD hotspots locate in east STC (**Figure 1B**). This pattern is not unexpected because the western STC is much more complex in topography and environmental heterogeneity than the eastern STC. Spatial environmental heterogeneity always has a strong positive effect on SD by allowing coexistence of species with fitness optima at different positions along an environmental gradient (Vellend, 2005) as Huang et al. (2012) indicated. However, the effects of environmental heterogeneity on genetic diversity are highly variable and context dependent, which means that genetic diversity does not necessarily increase with environmental heterogeneity (Vellend, 2005). Furthermore, if the total number of individuals in a locality is fixed, adding species to the community by increasing heterogeneity necessarily reduces the average population sizes of the component species (Vellend, 2005). This might also contribute to the lower genetic diversity in western than that in eastern populations of STC.

The relationship between latitude and WGD is unclear since sequence diversity is positively correlated with latitude, but the correlation does not hold between gene diversity and latitude. This relationship means that within-population genetic diversity distributes quite evenly along the north-south direction in subtropical China. This pattern is predictable because subtropical China have never been covered by ice sheets during the Quaternary glaciations (Shi et al., 1986). Most plants in this region survived in multiple refugia and have never been expelled to the south (see reviews in Qiu et al., 2011; Liu et al., 2012), thus the northern populations of STC may maintain relatively high genetic diversity.

### Relationship Between Genetic Diversity and Species Diversity

Intraspecific genetic diversity is difficult to measure at large scales (i.e., over large areas and for many species), a common solution to overcome these difficulties is to find a reliable surrogate for genetic diversity and the relationship between SD and genetic diversity has recently gained renewed interest (Kahilainen et al., 2014; Vellend et al., 2014). In this study, we found that AGD are positively associated with regional SD (**Table 4**), and nine of 14 WGD hotspots are also spatially congruent with the SD hotspots (**Table 2** and **Figure 2**). High genetic divergence among populations may accelerate the speciation, differentiation and preservation for the species living in these areas (e.g., Sturmbauer and Meyer, 1992). Moreover, higher divergence areas may also be expected in “suture zones” where formerly allopatric lineages of multiple species hybride (Remington, 1968). The hybrid zone has been shown to be a center of biodiversity (i.e., greater species richness and abundance) in many organism groups, such as insects and fungi (Whitham et al., 1994). Therefore, hybridization between divergent lineages generates new gene combinations that can contribute to ecological divergence and facilitate speciation and adaptive evolution in some cases (Arnold, 1997; Rieseberg et al., 2017).

Another component of genetic diversity (WGD), however, is negatively correlated with five SD indices in subtropical China (**Table 4**), except for sequence diversity and ER and PD. These results are at odds with the theoretical predictions that local habitat characteristics such as area, isolation, and spatial/temporal heterogeneity possibly induce parallel effects on species and genes via migration, drift and selection (Vellend, 2003, 2005). Empirical studies also found that correlations between SD and GD were generally positive (Vellend, 2003; Vellend and Geber, 2005). However, there are notable exceptions when environmental stochasticity is large and when species are rare (Vellend, 2005). As we noted before, high number of species in an area may result in small size of populations and thus creates low level of WGD. In addition, speciation (macroevolution) and differentiation (microevolution) resulting from processes acting at very different time-scales may yield unconnected or opposed levels and patterns of diversity at macroecological scales (Fady and Conord, 2010).

Overall, the relationships of WGD-SD and AGD-SD are inconsistent. In other parts of the world such as southern Europe, such pattern was also observed. In southern European forests, there is higher species richness than in northern Europe because more suitable environment existed during the Quaternary glaciations. However, chloroplast DNA variation in 22 widespread European trees and shrubs showed southern populations were more genetically divergent, but within-population diversity peaked north of the main mountain ranges such as Alps Mountains, rather than south of them (Petit et al., 2003). Our results support the hypothesis that the contribution of a population to total SD depends more on its divergence from other populations than on its intrinsic within-population diversity (Petit et al., 1998, 2003). However, our estimates of genetic diversity and SD are not taken from the same communities as SGDC studies routinely did, the significance of our results should be cautiously interpreted.

### Implications for Conservation

One of the most important reasons for rare consideration of genetic diversity in conservation prioritization is that intraspecific genetic diversity is difficult to measure at large scales (Taberlet et al., 2016). With the increased availability of molecular data, it is now possible to consider the genetic diversity of a large number of species simultaneously. In this study, we synthesized population genetic or phylogeographic datasets of 33 plant species into multi-species genetic landscapes in subtropical China, one of the most important ecoregions in the country. Obviously, this work relied on previous population genetic and phylogeographic studies, sampling more species representing different ecotypes and gathering genomic data representative of both functional and selectively neutral diversity are needed to provide genetic landscapes with greater resolution and reflect patterns of adaptive genetic diversity. In spite of this, the results of this study have at least three important implications for prioritizing conservation efforts in STC.

First, this study found that the western STC (Hengduan Mountains and Yunnan Plateau) is rich in among-population genetic diversity, possessing four major AGD hotspots. It is well known that the areas surrounding the east and southeast of Qinghai-Tibetan Plateau (QTP) comprise one of the key high-latitude biodiversity hotspots in the world (Myers et al., 2000). The uplift of the QTP created a large altitudinal gradient across the region (Wu, 1987) and the alteration of topography and past climatic changes associated with mountain uplifts can cause fragmentation of species distributions, which can lead to reduced gene flow between isolated populations (Yuan et al., 2008). This process initiates allopatric divergence that can ultimately drive populations toward speciation (Mayr, 1963; Rice and Hostert, 1993). Our results strengthen that the western STC should be put a high conservation priority not only for its exceptional species richness and habitat vulnerability, but also for its high among-population genetic diversity that has generated a cradle of seed plant biodiversity (Lu et al., 2018).

Second, we found that WGD is positively correlated with longitude, with most WGD hotspots locate in east STC. This pattern suggests that eastern STC may represent an intraspecific genetic reservoir. However, protected areas in eastern STC are fragmented, largely as a result of urbanization and administrative division (Lu et al., 2018). Establishing more connections between existing nature reserves and national parks that span provincial borders to facilitate gene flow and thus prevent the loss of intraspecific genetic diversity due to genetic drift and inbreeding in isolated populations is urgently needed.

Third, the importance of intraspecific diversity for ecosystem functioning and for preserving the evolutionary potential of species demands that genetic diversity should not be neglected when designing conservation strategies and networks of protected areas (Laikre et al., 2009). However, landscape level spatial genetic information for multiple species are still mostly lacking, an effective approach would be to predict patterns of intrapopulation genetic diversity based on the SGDCs (Kahilainen et al., 2014). In this study, we found a positive and significant correlation between AGD and SD, but we failed to find such a relationship between WGD and SD. Obviously, spatial patterns of species richness in plants in subtropical China cannot generally be used as surrogate for their intraspecific diversity, at least for within-population genetic diversity. This situation implies that more efforts should be invested into the surveys of genetic diversity of plants in subtropical China to refine our assessment of evolutionary hotspots, especially by applying a standard sampling (e.g., Eidesen et al., 2013) and genomic data representative of both functional and selectively neutral diversity. Fortunately, the current revolution in DNA sequencing technology and rapidly developing transportation infrastructure in China will permit large-scale evaluation of genetic diversity, and consequently allow a better implementation of the Convention of Biological Diversity (CBD) by integrating intraspecific genetic diversity into conservation projects of subtropical China.

## Author Contributions

DF and ZZ conceived this study and wrote the manuscript. JH, ZS, and SC analyzed the data. DF, HH, and YK drew the figures. All the authors read and approved the final manuscript.

## Conflict of Interest Statement

The authors declare that the research was conducted in the absence of any commercial or financial relationships that could be construed as a potential conflict of interest.
